# Heart-lung transplantation in the United Kingdom: Trends and outcomes over 4 decades

**DOI:** 10.1016/j.jhlto.2026.100572

**Published:** 2026-04-23

**Authors:** Miguel Angel Reyes Roque, Emre Erturk, Sally Rushton, Aaron Ranasinghe, Helen Spencer, Alexandra Ball, Debra Thomas, Jasvir Parmar, Vasiliki Gerovasili, Louise Coats, Andrew J. Fisher, Louise A. Kenny

**Affiliations:** a Statistics and Clinical Research, NHS Blood and Transplant, Bristol, UK; b Adult Congenital and Paediatric Heart Unit, Freeman Hospital, Newcastle Upon Tyne Hospitals NHS Foundation Trust, Newcastle Upon Tyne, UK; c Population Health Sciences Institute, Newcastle University, Newcastle Upon Tyne, UK; d Institute of Transplantation, Newcastle Upon Tyne Hospitals NHS Trust, Newcastle Upon Tyne, UK; e Translational and Clinical Research Institute, Newcastle University, Newcastle Upon Tyne, UK; f Cardiac Surgery, Queen Elizabeth Hospital, University Hospitals Birmingham NHS Trust, Birmingham, UK; g Respiratory Medicine and Cardiothoracic Transplantation, Great Ormond Street Hospital, London, UK; h The Transplant Centre, University of Manchester NHS Foundation Trust, Manchester M23 9LT, UK; i The Ex-Vivo Lab, Division of Cell Matrix and Regenerative Medicine, School of Biological Sciences, Faculty of Biology, Medicine and Health, The University of Manchester, Manchester Academic Health Science Centre, Manchester M13 9NT, UK; j Department of Transplantation, Royal Papworth Hospital NHS Foundation Trust, Cambridge Biomedical Campus, Cambridge, UK; k Transplant Department, Royal Brompton and Harefield Hospitals Guy's and St. Thomas' National Health Service Foundation Trust, London, UK; l Imperial College London, Faculty of Medical Sciences, London, UK

**Keywords:** Transplantation, Heart-lung transplantation, Congenital heart disease, Pulmonary hypertension, Donor, Recipient

## Abstract

**Background:**

Combined en-bloc heart-lung transplantation (HLT) remains the optimal treatment for selected patients with end-stage cardiopulmonary disease. The indications for and utilization of HLT have changed significantly over time. We report the changing landscape of HLT in the UK over recent decades, identifying factors that may inform future organization of services.

**Methods:**

Data were extracted from the UK Transplant Registry spanning 1984 to December 2023. All patients who were registered for and/or underwent HLT were included in our analysis. Baseline characteristics and outcomes from listing and transplantation were compared between 2 eras: pre-2000 and post-2000.

**Results:**

Pre-2000, 1,199 patients were registered for HLT, and 915 received HLT, post-2000, there were 473 registrations and 200 transplantations, reflecting a significant decline in the use of this procedure. Those listed post-2000 were older, more symptomatic, and with more prior cardiac surgeries. Cystic fibrosis, the leading indication pre-2000, declined substantially in the later era. For patients requiring HLT in the later era, the risk of dying on the waiting list exceeded the chance of receiving a transplant. Median post-transplant survival improved from 4.07 (IQR 0.2, 12.3) to 7.9 (IQR 0.8, 21.6) years.

**Conclusion:**

Although HLT activity has declined, a considerable need remains, particularly among patients with congenital heart disease and pulmonary hypertension. The changes in patient demographics and high waiting list mortality highlight systemic inefficiencies in organ allocation and underscore the need for a revised system to ensure timely and equitable access for this high-risk population.

## Background

The first successful heart-lung transplantation (HLT) was performed by Reitz and Shumway at Stanford, USA, in 1981 in a patient with pulmonary arterial hypertension (PAH), marking a significant milestone in the treatment of end-stage cardiopulmonary disease.^1^ Over the subsequent 4 decades, however, the emergence of new treatment strategies for cystic fibrosis (CF) and idiopathic pulmonary artery hypertension (IPAH), along with earlier diagnosis and surgical management of congenital heart disease (CHD), has led to changing indications and reduced utilization of HLT across the globe. Additionally, improvements in single organ transplantation mean that en-bloc HLT has been replaced by either bilateral lung transplant (BLT) or orthotopic heart transplant (OHT) for most patients and is preferred where possible to optimize donor organ utilization.^2^, ^3^ Although HLT continues to be the optimal management strategy for patients with a specific set of clinical features, it is now applicable to only a small group of patients and has become a rarely performed operation.^4^

HLT is presently reserved for those with irreversible dual end-stage organ failure: either a primary lung condition complicated by severe, refractory ventricular failure or a primary cardiac condition (typically CHD) with irreversible PAH.^5^ Candidates free from complex CHD or left ventricular compromise or those with surgically correctable cardiac conditions (eg coronary artery disease, valvular disease) can achieve comparable outcomes with BLT combined with corrective cardiac surgery.^2^, ^6^, ^7^, ^8^, ^9^ BLT has become the preferred strategy for the majority of patients, even with severe right ventricular dysfunction, due to the now well recognized recoverability of the right ventricle following transplant.^9^, ^10^ Even those with Eisenmenger syndrome in the setting of atrial septal defect (ASD) demonstrate reversibility of severe right ventricular dysfunction and tricuspid regurgitation with better survival following BLT with concurrent ASD closure than HLT.^11^ There remains a small group with uncorrectable heart defects and/or significant left ventricular dysfunction who are dependent upon HLT for survival.

Advances such as the use of lungs and hearts from donation after circulatory death, ex-vivo lung perfusion transplant and ex-situ perfusion have greatly expanded the donor pool for hearts and lungs for single organ transplants, reducing waiting list times across the UK, Europe and the USA.^12^, ^13^, ^14^, ^15^ Despite attempts to apply these technologies to multi-organ transplant, it has not been possible to perform normothermic ex-vivo perfusion on a heart-lung block.^16^ As a result, those waiting for HLT continue to be disadvantaged and are more likely to die before reaching transplant than individuals listed for single organ transplant.^17^

Recipient selection criteria and organ allocation for HLT varies markedly across Europe and the US due to disparities in healthcare systems, donor organ availability, and disease prevalence.^18^, ^19^, ^20^ In the UK, the National Health Service (NHS) operates under strict guidelines that govern which patients can be considered, emphasizing comprehensive evaluation of specific measures of pulmonary and cardiac function.^20^, ^21^, ^22^ Evolving policies for single-organ transplant has led to the development of routine, urgent (heart 1999, lung 2017), and super-urgent (heart 2016, lung 2017) listing criteria. Patients are listed routinely for HLT when criteria are met without additional external review. For a routinely listed patient to be allocated organs, both heart and lungs must have been offered and declined through the super-urgent and urgent separate heart-only and lung-only waiting lists. Urgent HLT listing was formalized in 2022 and requires national agreement from an NHS organ allocation panel for eligibility. A patient on the urgent HLT list is allocated in-line with heart-alone recipients, with the lungs prioritized to this patient over urgent but not super-urgent lung-only recipients.^23^, ^24^ Super-urgent HLT listing is currently not formalized but has been utilized in ad hoc cases. The competition for organs from urgently listed patients for heart-only or lung-only dramatically affects access to HLT for those listed for this procedure.

Outcomes for HLT have improved significantly due to immunosuppression, surgical technique, recipient and donor selection, and peri-operative management, including mechanical circulatory support (MCS) and extracorporeal membrane oxygenation^25^, ^26^). Early mortality continues to be a concern despite improvements, with 1-year survival reported between 66.6% and 89%^4^, ^27^ [ISHLT].^28^ Advanced donor age, need for recipient MCS or previous surgery, and center transplant volumes are all factors that impact survival.^25^ Despite high early mortality and lower median survival than single organ transplant (HLT:3.3, BLT:7.1, OHT:10.4 years), ISHLT registry data demonstrates that conditional median survival for all patients who survive to 1 year is comparable to single organ transplant (HLT: 10.0, BLT: 9.7, OHT: 13.0 years).^29^

Internationally, CHD is now the leading indication for HLT, and as such, it is important to recognize that this group have comparable long-term conditional survival if they survive the first year to those undergoing single-organ transplantation^27^ [ISHLT].^29^ As pathology and indications for HLT change and organ allocation policies evolve, it is imperative to monitor this diminishing patient group to ensure they are not disadvantaged as they continue to rely on HLT as a life-saving treatment. The aim of this study is to describe the changing landscape of HLT in the UK over 4 decades and identify factors that may inform future practice.

## Materials and methods

### Study population

#### Registration cohort

Data were extracted from the UK Transplant Registry (UKTR) on 10 September 2024 for all patients (adult and pediatric) ever simultaneously registered for heart and lung transplant (HLT) in the UK up to 31 December 2023. In the UKTR, a registration is defined as a period of activation on a transplant waiting list ending in either removal from the list, death on the waiting list, or transplant. Clinical data capturing detailed characteristics of the patient at the time of registration was introduced in the UKTR in 1995. Only basic demographic data were available prior to this year. If there was no clinical data available at registration, the closest record within 5 years was accepted.

#### Transplant cohort

Patients from the HLT registration cohort who underwent HLT together with those who received HLT via another route (heart only registration, lung only registration, or no prior HLT registration on the UKTR) formed the transplant cohort. Of note, registration only commenced in 1987; however, HLT commenced in 1984. Recipient, donor, and operative data were extracted for these patients as well as post-transplant survival.

#### Statistical analysis

Baseline characteristics of registration and transplant cohorts were compared pre-2000 (excluding the year 2000) and post-2000 (including the year 2000). The year 2000 was selected as the era cutoff, as there was an appreciable change in the number of registrations and transplants at this time. The Chi-squared test was used to compare categorical variables. Continuous variables were compared by the independent Student *t*-test if normally distributed and the Wilcoxon rank sum test if non-normally distributed. Registration outcomes were analyzed using a competing risks framework, where unadjusted cumulative incidence functions were compared using Gray’s test. For analysis of time to registration outcome, day zero was the first date a patient was active for both HLTs (so if a patient had a prior period of being active for just 1 organ, this time was discounted). If a patient was removed from 1 transplant list but remained active on the other, the patient was classed as removed. However, if a patient remained active for both organs up to the point of transplant but only received 1 organ, the outcome was “transplanted - not HLT.” If a patient was removed due to deteriorating condition, this was classed as death. For a patient with multiple heart-lung registrations, all registrations were combined to describe the complete journey. The Kaplan-Meier method was used to analyze time from first HLT to death, where survival curves were compared using the log-rank test. Due to a large amount of missing data in the early era, all analyses were unadjusted.

#### Ethics and data management

Data analysis was performed by National Health Service Blood and Transplant, which maintains the UK Transplant Registry on behalf of UK transplant centers. Under the UK General Data Protection Regulation, National Health Service Blood and Transplant are permitted to analyze patient data without additional patient consent. All data management and analysis were carried out using SAS 9.3 (SAS Institute, Cary, NC).

## Results

**Figure 1:**
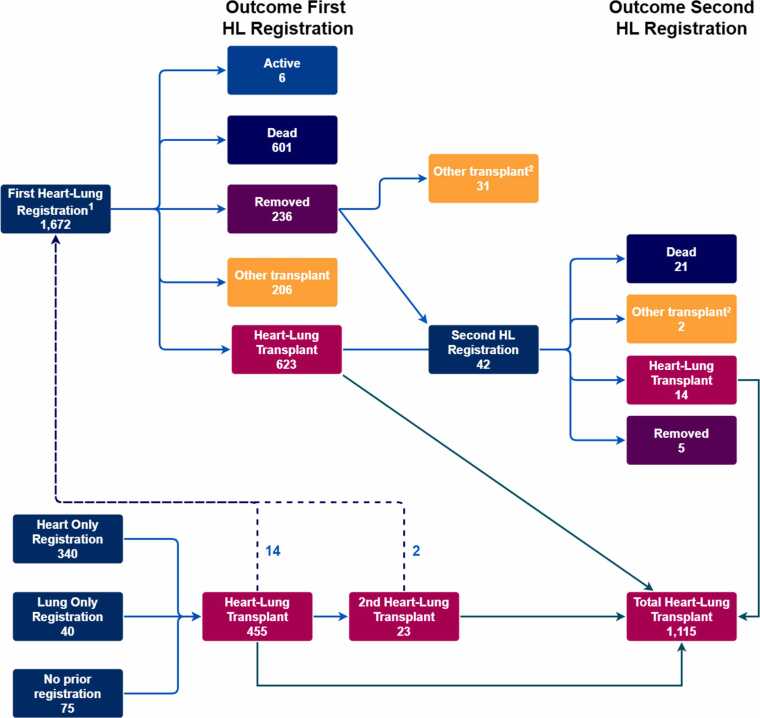
Schematic *diagram* showing the journey of those listed for or undergoing heart-lung transplant (1987-2024) [^1^Of those formally registered for heart-lung transplant (commencing in 1987): 28 patients had previous heart-only registration, and 7 had previous lung only registration; 1 patient had a previous heart-only transplant and 5 had previous lung-only transplant, ^2^Of those undergoing transplant other than heart lung following formal registration: a total of 194 lung-only transplants, 29 heart-only transplants, and 15 multi-organ transplants were performed: including heart-kidney (1), heart-lung-kidney (1), heart-lung-liver (11), and lung-liver (1)].

The clinical practice of HLT commenced in 1984, while formal registration for the HLT waiting list commenced 3 years after surgical commencement in 1987. From 1987 to 2023, 1,672 patients were identified as ever having been registered for HLT. From 1984 to 2023, 1,115 patients underwent HLT in the UK. Figure 1 provides a schematic summarizing the transplant journey of these cohorts. 43% (*n* = 455) of HLT recipients were not registered for heart-lung but arrived at this type of transplant via an alternative route; 340 were registered for heart-alone transplant, 40 for lung-alone transplant, and 75 were unregistered on the UKTR (occurring as late as 2013). HLT via other routes reduced over time but continued until 2017.

## Combined heart-lung registration

### Population characteristics

**Figure 2:**
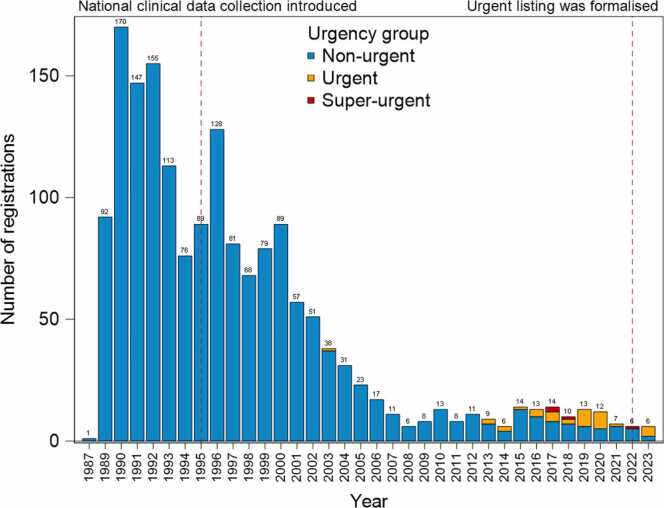
Number of formal registrations for heart and lung transplant (commencing in 1987) by year and urgency. Urgent heart-lung registration was introduced in 2022 (dotted line). Prior to this, patients could be listed for urgent heart and lungs on both lists, as seen on the graph. Those indicated as super-urgent have been listed super-urgently on both heart alone and lung alone lists with special consideration following national panel discussion.

**Figure 3:**
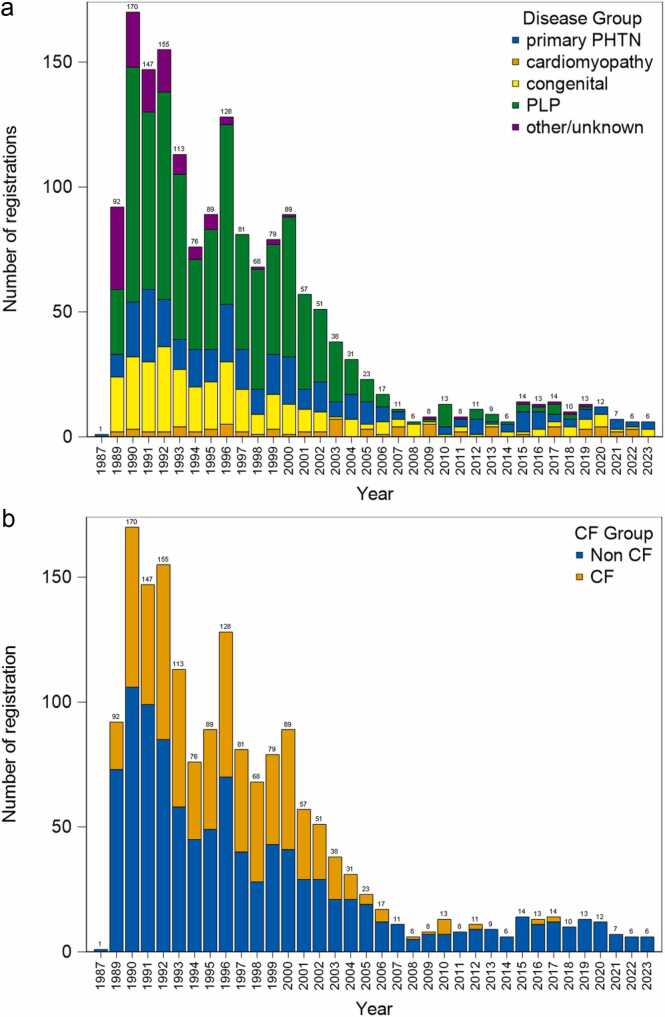
**(a)** Underlying conditions of those formally registered for heart-lung transplantation between 1987 and 2023 and **(b)** cystic fibrosis versus all other conditions registered for heart-lung transplantation.

1,672 patients were identified on the UKTR as ever having been registered for HLT. The earliest registration date was in 1987, and the cut-off date was December 2023. 1,199 patients (71.7%) were registered pre-2000 and 473 patients (28.3%) were registered post-2000 (Figure 2). Registrations for HLT steadily diminish over the study period until 2000, when numbers stabilize and consistently remain fewer than 15 cases nationally per year. Of the 1,672 patients, 42 had more than 1 heart-lung registration, with the maximum number of registrations being 2. Baseline demographics of those registered for HLT are shown in Table 1. Those registered in the later era (2001-2023) were older and more symptomatic as measured by NYHA class, with more previous heart surgeries and implantable cardiac devices (*p* < 0.01 for all comparisons). This latter cohort have typically undergone conventional medical and surgical management unavailable in the previous era, rendering them older with advancing disease and more surgically complex at the time of registration. The indications for HLT have changed substantially over the study time frame. Primary lung pathology, in particular CF, was the major indication pre-2000, with numbers of cases falling rapidly during the second era (Figure 3). In the last 5-years there has been just 1 registration out of a total of 44 for primary lung pathology, with conditions leading to registration for HLT comprised primarily PAH (36%), CHD (32%) and cardiomyopathy (27%).

**Table 1:** Baseline Characteristics of Individuals Registered for Heart Lung Transplant Between 1987-1999 and 2000-2023

	1987-1999 (*n* = 1199)	2000-2023 (*n* = 473)	*p*
Male	562 (46.95%)	196 (41.44%)	0.04
*Missing*	*2*	*0*	
Age Median (IQR)	26 (19-38)	32 (21-47)	<0.0001
*Missing*	*1*	*0*	
Pediatric patients (<16 years)	203 (16.9%)	71 (15.0%)	0.34
*Missing*	*1*	*0*	
Disease group Cardiomyopathy	29 (2.5%)	48 (10.2%)	<0.0001
Congenital	237 (20.8%)	84 (17.8%)	
Idiopathic Pulmonary Hypertension	185 (16.3%)	126 (26.7%)	
Primary Lung Pathology	634 (55.7%)	207 (43.9%)	
Other	53 (4.7%)	7 (1.5%)	
*Missing*	61	1	
Home oxygen^1^*N* (%)	250 (61.7%)	261 (55.7%)	0.07
*Missing*	*794*	*4*	
NYHA^1^ I	3 (0.8%)	3 (0.7%)	<0.0001
II	53 (14.2%)	37 (8.1%)	
III	236 (63.1%)	251 (55.2%)	
IV	82 (21.9%)	164 (36.0%)	
*Missing*	*825*	*18*	
One or more previous open-heart surgeries^1^	37 (9.1%)	68 (14.53%)	0.01
*Missing*	*793*	*5*	
Previous thoracotomy^1^	32 (7.9%)	38 (8.12%)	0.91
*Missing*	*794*	*5*	
AICD^1^	2 (0.5%)	21 (4.49%)	0.0002
*Missing*	*793*	5	
In hospital at registration^1^	73 (18.0%)	80 (16.99%)	0.69
*Missing*	*793*	*2*	

Baseline characteristics of individuals registered for Heart Lung Transplant between 1987-1999 and 2000-2023.

1 Only available from 1995 (*N* = 445 in 1987-1999 cohort). NYHA, New York Heart Association; AICD, Automatic Implantable Cardiac Defibrillator; IQR, Inter Quartile Range.

### Outcomes after registration for HLT

**Figure 4:**
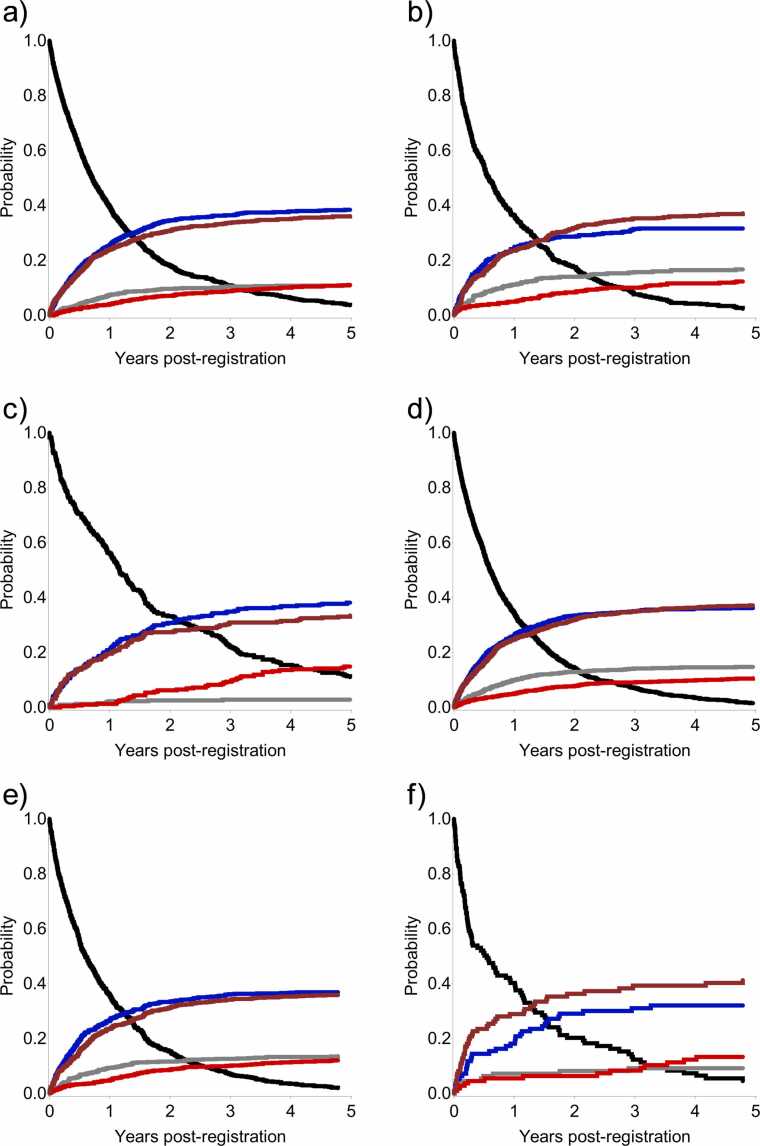
Competing interests' curves showing outcomes following formal heart-lung transplantation registration between 1987 and 2023 by era (a) pre-2000 (b) post-2000, by pathology (c) recipient congenital heart disease (d) recipient all other disease and by previous surgery (e) recipient without previous thoracotomy or sternotomy and (f) recipient with previous thoracotomy or sternotomy [HLT: heart lung transplant].

The waiting list mortality of all patients registered for HLT was 37% (*n* = 622). Other outcomes, including other transplant or removal from the list, are depicted in Figure 1. The competing risks of different outcomes following listing for HLT are shown in Figure 4. Pre-2000, the probability of a registered patient receiving HLT within 5 years was 38.4%, whereas post-2000, this dropped significantly to 31.6% (*p* = 0.0319, Figure 4a and b). Furthermore, in the later era, in contrast to pre-2000, if still waiting 18 months after registration, the probability of dying on the waiting list starts to exceed the probability of receiving a HLT. In the later era, the risk of dying on the waiting list is 37.0% and the risk of receiving a HLT is 31.6%.

Patients with CHD registered for combined organs were as likely to receive HLT as those with non-congenital pathology (38.1% vs 36.2% within 5-year, respectively, *p* = 0.8757), however those with non-congenital pathology were more likely to receive a single organ transplant (Figure 4c and d, 14.8% vs 2.8% within 5 years, respectively, *p* < 0.0001). Some non-CHD patients become suitable for single organ transplant with medical therapy, a path that is rarely available to those with CHD. Those patients registered for HLT with a history of previous surgery are more likely to die whilst waiting than receive HLT (Figure 4e and f, 41.4% died, 32.1% received HLT). To understand the contemporary situation for UK patients registered for HLT, we reviewed the outcomes from registration over the last 10 years (2014-2023). During this period, 101 patients were registered for HLT. Median waiting time to HLT regardless of urgency was 1017 days (95% CI:583-1451). For those urgently listed, median waiting time was 346 days (95% CI:0-748). Of these 101 patients, 35 (35%) died whilst waiting.

## Combined heart lung transplants

### Population characteristics

**Figure 5:**
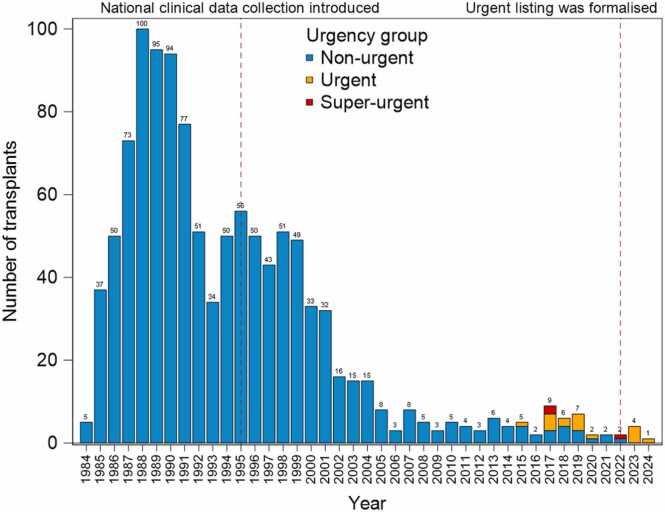
*Number of combined heart-lung transplants by year and urgency* (comprising those with simultaneous heart-lung registration as well as those registered for heart-alone, lung-alone, or not registered). Formal registration commenced in 1987.

The UKTR contained records of 1,115 patients who underwent HLT. The earliest transplant date was in 1984 and the latest in 2024. 915 patients were transplanted pre-2000, and 200 patients were transplanted post-2000 (Figure 5). 33 patients had multiple HLT (32 patients underwent 2 HLTs and 1 patient underwent 3). Baseline demographics of those undergoing HLT are shown in Table 2.

**Table 2:** Baseline Characteristics of Recipients Undergoing Heart-Lung Transplantation Between 1984-1999 and 2000-2024

	1984-1999 (*n* = 915)	2000-2024 (*n* = 200)	*p*
Graft number First HLT	883 (96.5%)	198 (99.0%)	
Second HLT	31 (3.4%)	2 (1.0%)	
Third HLT	1 (0.1%)	0 (0%)	
*Missing*	*0*	*0*	
Male	423 (46.3%)	80 (40.0%)	0.106
*Missing*	*20*	*0*	
Age (years) Median (IQR)	28 (18-39)	33 (24-46)	<0.0001
*Missing*	*10*	*0*	
Pediatric patients (<16 years)	169 (18.5%)	21 (10.5%)	0.006
Disease group Cardiomyopathy	16 (2.21%)	13(6.53%)	0.0005
Congenital	189 (26.07%)	44 (22.11%)	
Idiopathic pulmonary hypertension	156 (21.52%)	57 (28.64%)	
Primary Lung Pathology	294 (40.55%)	77 (38.69%)	
Other	70 (9.66%)	8 (4.02%)	
Missing	190	1	
One or more previous open-heart surgeries^1^	16 (8.99%)	20 (10.42%)	0.64
*Missing*	*737*	*8*	
Previous thoracotomy^1^	14 (7.87%)	13 (6.77%)	0.68
*Missing*	*737*	*8*	

Baseline characteristics of recipients undergoing heart lung transplantation between 1984-1999 and 2000-2024.

1 Only available from 1995. HLT, heart lung transplantation; IQR, Inter Quartile Range.

### Donor Group

Baseline demographics of HLT donors are shown in Table 3. Pre-2000, donors are significantly older with a higher percentage of intracranial pathology, and there are fewer pediatric and trauma donors (*p* < 0.007 for all comparisons).

**Table 3:** Baseline Characteristics of Heart-lung Transplantation Donors Between 1987-1999 and 2000-2023

	1987-1999 (*n* = 915)	2000-2023 (*n* = 200)	*p*
Donor sex (male)	484 (54.1%)	73 (36.5%)	0.001
*Missing*	20	*0*	
Donor age (years) Median (IQR)	20 (13-32)	34.5 (21-47)	<0.0001
*Missing*	*10*	*0*	
Pediatric donors (<16 years)	302 (33.4%)	28 (14.0%)	0.007
*Missing*	*10*	*0*	
Donor cause of death Intracranial	355 (38.8%)	144 (72.0%)	<0.0001
Trauma	451 (49.3%)	28 (14.0%)	
Other/Not reported	109 (11.9%)	28 (14.0%)	

Baseline characteristics of heart lung transplantation donors between 1987-1999 and 2000-2023. IQR, Inter Quartile Range.

### Outcomes from transplantation

**Figure 6:**
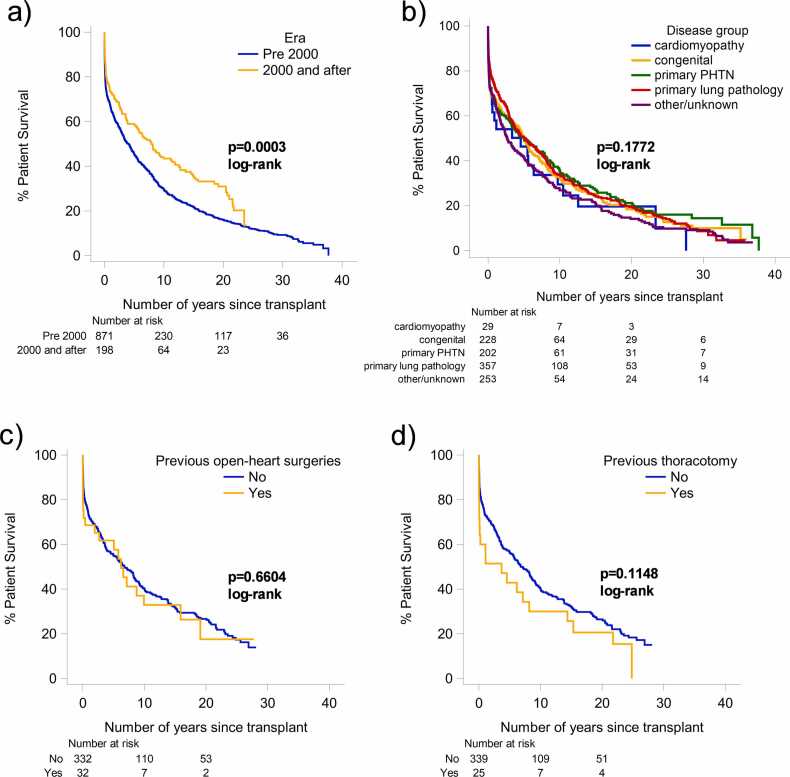
Kaplan-Meier survival curves showing difference in survival by (a) era of heart lung transplantation, (b) underlying condition (c) history of previous cardiac surgery, and (d) history of previous thoracotomy. [PHTN: pulmonary hypertension].

Survival analysis was performed on a per-patient basis, and the 1,115 HLTs corresponded with 1,081 unique patients (accounting for the patients who received multiple HLT). 12 patients were additionally removed due to missing survival data/previous single organ transplant; thus, this analysis comprised 1,069 patients. Kaplan-Meier curves are shown in Figure 6. Pre-2000, median survival time following HLT was significantly shorter at 4.07 years (IQR:0.2, 12.3) compared to 7.9 years (IQR:0.8, 21.6) for those transplanted post-2000 (*p* = 0.0003 log-rank, unadjusted hazard ratio = 0.709). In era 1, survival was 82.5% (95% CI:79.9-84.9), 66.9% (63.7-69.9), 45.5% (42.2-48.8), and 29.5% (26.5-32.7) at 30 days, 1 year, 5 years and 10 years, respectively. In era 2, survival was 88.9% (95% CI:83.6-92.5), 73.5% (66.7-79.1), 58.9% (51.6-65.5) and 43.4% (36.0-50.6) at 30 days, 1 year, 5 years and 10 years respectively demonstrating an improvement in both short and long-term survival (*p* = 0.03, 0.057, 0.001, and 0.0005, respectively). 5-year conditional survival (conditional on survival to 1-year) improved from 68% (95% CI:64.0-71.7) to 80.2% (72.4-85.9) from era 1 to era 2 (*p* = 0.008). Eight patients have survived beyond 30 years, with the maximum survival being 37.7 years.

No statistical difference in median survival was seen between indications for HLT, reflecting a susceptibility amongst all groups requiring HLT to the higher early mortality associated with this procedure. Median survival for those with CHD was 5.0 years (IQR: 0.2-15.2) compared to 4.6 years (0.2-10.5) for those with cardiomyopathy and 5.1 years (0.2-17.0) for those with idiopathic PAH (*p* = 0.1772). Survival for those with CHD was 80.7% (95% CI: 75.0-85.3), 67.9% (61.4-73.5), 49.7% (42.9-56.0), 31.8% (25.7-38.1) at 30 days, 1 year, 5 years, and 10 years respectively. Survival for those with cardiomyopathy was 82.8% (95% CI:63.4-92.4), 57.9% (37.9-73.5), 46.3% (27.3-63.4), 29.5% (13.6-47.4) at 30 days, 1 year, 5 years and 10 years respectively. Survival for those with IPAH was 83.2% (95% CI: 77.3-87.7), 64.9% (57.8-71.0), 50.1% (43.0-56.8), 35.6% (28.9-42.4) at 30 days, 1 year, 5 years, and 10 years, respectively.

No difference was seen between those with and without previous history of heart surgery (log-rank *p* = 0.6604), and although those with prior thoracotomy had worse median survival: 3.7 years (IQR:0.5-15.3) than those without: 6.8 years (0.8-20.5), this did not reach statistical significance (log-rank *p* = 0.1148). No difference was seen according to time on the waiting list (*p* = 0.7039 log-rank). Non-urgent and urgent patients showed no difference in survival post HLT (*p* = 0.4940 log-rank). Only 4 patients were listed under the panel agreed super-urgent listing, and only 3 patients received an HLT from this status (*n* = 1 received a heart-only from the super-urgent listing). Two super-urgent HLT were transplanted from mechanical support. These 3 patients of mixed pathologies: cardiomyopathy non congenital (1), IPAH(1), other(1)] had a 33% 30-day mortality. It is difficult to comment upon this small group.

## Discussion

Over the past 40 years, HLT has emerged as an effective treatment for selected patients with combined end-stage heart and lung disease. Concurrent parallel advances in medicine have seen significant changes in the need for HLT and the indications for its use. Today HLT has become a rare operation, but critically, there remains a patient group for whom this procedure is the only viable route to survival and longevity.

Trends in HLT in the UK reflect the consequences of advances in medicine alongside growing expertise in transplantation. CF, a key indication in the early era, has all but disappeared latterly with the successful use of BLT alone and the advent of effective medical management through CF transmembrane conductance regulator (CFTR) modulator therapies.^30^ In the last 5 years of HLT in the UK, an equal distribution of cardiomyopathy, PAH, and CHD are observed as the key underlying indications. In most centers worldwide, BLT has become the preferred strategy for patients with PAH and CF, demonstrating better survival [median 9.9 years] and equivalent outcomes^31^, ^32^, ^33^, ^34^, ^35^, ^36^ [ISHLT]. Right ventricular (RV) dysfunction or severity of tricuspid regurgitation is not preclusive, as rapid RV recovery is noted post-transplant.^10^, ^37^, ^38^, ^39^ Additionally, BLT can also be performed with concurrent cardiac repair of simple defects such as ASD and lead to good outcomes with RV recoverability.^11^ In the future, HLT is likely to be reserved for the growing number of adults with complex CHD and a smaller number with severe PAH and left ventricular dysfunction where no other options exist.

HLT recipients in our cohort are increasingly older and sicker with more complex surgical histories, including more prior sternotomies and thoracotomies. This reflects the growing ability to manage underlying conditions with conventional means whilst also better balancing the deteriorating symptom burden with expectations of post-transplant survival. Unlike the United States and other European countries where listing criteria offer more flexibility, in the UK, patients are required to meet stringent symptomatic criteria for transplant listing.^21^, ^22^ Donors allocated to HLT recipients are also increasingly older with less trauma and more intracranial pathology as the cause of donor death. HLT is associated with higher early mortality, and our results reflect this; however, in keeping with ISHLT data, early survival at 1-year improved over the 2 eras from 66.9% to 73.5%.^25^, ^27^ Despite increasing complexity of donor and recipient, median survival improved from 4.1 (IQR: 0.2, 12.3) pre-2000 to 7.9 years (IQR: 0.8, 21.6) post-2000. This improvement is likely multifactorial, resulting from a combination of optimized peri-operative care, advancing surgical technical ability, monitoring and management of rejection, immunosuppression, donor-recipient selection, and use of MCS.

While others have found favorable outcomes with CHD patients reporting 93% survival at 30 days, 51% at 5 years, and 43% at 10 years, early survival for CHD patients undergoing HLT in the UK are lower in this study at 80.7% at 30 days, 67.9% at 1 year, 49.7% at 5 years and 31.8% at 10 years.^40^ It is important to note that survival for CHD patients undergoing HLT is comparable to those with other diagnoses in both our cohort and ISHLT data despite the additional complexities.^4^, ^27^, ^41^ HLT has been shown to offer better survival than BLT in the setting of Eisenmenger syndrome, particularly where the primary etiology is a ventricular septal defect.^11^, ^42^ Survival conditional to 1 year, for those undergoing HLT with and without Eisenmenger is reported as 11.3 and 13.3 years, respectively, which is comparable to OHT (13 years) and better than BLT (9.7 years).^3^ Our findings, along with the experience of others reinforces the effectiveness and safety of HLT as a viable treatment option for CHD patients who are often perceived as too complex to be eligible by a range of clinicians.

Previous surgery and its association with dense adhesions and collaterals increases peri-operative bleeding risk and is acknowledged by transplant surgeons to be a factor determining clinical outcomes. Whilst our data does not demonstrate the significance of this factor, we found a trend toward higher early mortality in those HLT recipients with previous thoracotomy. Pre-operative cross-sectional imaging, pro-active embolization of large aorto-pulmonary collaterals and incorporation of CHD surgical expertise (where redo sternotomy is routine practice) into the transplant team may mitigate some of this risk. Patients with previous surgeries also present an immunological challenge. CHD patients, in particular, may have undergone multiple procedures involving cardiopulmonary bypass and often carry transfusion related antibodies that limits their donor pool.

HLT patients have long waiting times. Over the last decade in the UK, median waiting time for HLT was 1,017 days, compared to 867 days for non-urgent OHT, or 530 days for non-urgent BLT. Even those urgently listed with higher risk of death had a median wait for HLT of 346 days, compared to 43 days for urgent OHT and 22 days for urgent BLT.^43^ This substantial discrepancy in waiting time highlights the disadvantage placed upon those needing HLT and questions the validity of an urgent list upon which patients wait an average of a year. This contrasts with Europe, where others have found no difference in waiting time for a graft.^9^, ^20^ For example, in France, which has a similar healthcare systems to the UK (with state funding and almost-universal health coverage), better access to organs with shorter waiting times has been observed, for example, in pulmonary hypertension. This difference may relate to the complexity of patients on the list or greater centralization of transplant services.^20^

Congenital patients, who will soon form the bulk of those listed, inevitably wait longer due to the factors described and are thus further disadvantaged, despite having equivalent and in some circumstances better outcomes. Unlike single organ transplantation, patients requiring urgent HLT are required to be discussed and agreed at a national panel. If agreement is met, access to organs is then further limited by prioritization of super-urgent single organs—a category not yet formally accessible for HLT recipients. The consequence of this disparity in waiting times is the waiting list mortality, where a patient requiring HLT has a 35% chance of dying before reaching transplant compared with someone requiring non-urgent OHT (13%), non-urgent BLT (26%), urgent OHT (9%) or urgent BLT (19%).^43^

Perhaps patients waiting for HLT are sicker and more difficult to support, rendering them more vulnerable to death whilst waiting, but the more credible explanation is that present listing criteria prioritize single-organ recipients disproportionately and fail to consider the nuances of the patient’s underlying condition, which may further exacerbate inequity. As HLT increasingly becomes confined to a small group of anatomically and medically complex recipients, expertise should be focused in dedicated multi-disciplinary teams. With such low volumes of HLT in the UK, national knowledge sharing and education is key to avoid even longer waits and to maintain outcomes. Under-referral driven by unfamiliarity with HLT and risk-averse practice may disadvantage eligible patients, highlighting the need for clear referral guidance.

Donor organ scarcity inevitably drives utilitarian discussions that favor single-organ transplantation as a means of achieving “the greatest good for the greatest number.”^44^ However, applying this principle by prioritizing 2 single-organ transplants over 1 dual-organ procedure oversimplifies the complexity of transplant value. Patients requiring HLT demonstrate survival outcomes that are comparable to, and in some cases better than, those receiving isolated heart or lung transplants. Equity in allocation must therefore consider not only transplant numbers but also quality of life, years of life gained, and the socioeconomic impact of recovery. These patients deserve equitable access to evaluation and listing, guided by outcome data rather than the widespread belief that allocating 2 single organs is inherently more beneficial than 1 combined transplant.

### Future UK considerations

To ensure equitable access to transplantation for patients requiring HLT, we advocate for 2 key policy actions. First, formalize a super-urgent listing process for HLT to ensure that critically ill patients can be assessed within a nationally agreed framework to ensure consistency of listing practice.

Second, a system-wide review of allocation frameworks is needed to ensure that both single and dual-organ transplant candidates are evaluated using consistent, evidence-based criteria. This should include not only clinical urgency and projected outcomes but also broader indicators of value—such as quality of life, cost-effectiveness, and social contribution—reflecting the NHS’s responsibility to deliver fair and meaningful benefit across the population.

### Limitations

The retrospective nature of this study presents challenges in capturing the political, societal, and clinical changes that have impacted on the evolution of HLT over almost 4 decades. While we highlight the major policy changes, which we feel have affected HLT patients, there are of course the more subtle changes in clinical practice which drive referral and transplant patterns and are not easily characterized. Data completeness is limited, particularly in the early era of transplantation, which impacts on the accuracy with which we can describe this early cohort of patients and make robust comparisons with the later era. Future work must focus on the remaining patients who rely on HLT in the present day. We should consider whether present data collection should be further refined to best describe this cohort’s issues and enable continuing evolution and optimization of listing criteria, recipient selection, and risk stratification and finally further improve outcomes.

## Conclusions

The last 40 years have seen changing disease indications and advances in transplantation delivery that have underpinned a decline in the prevalence of HLT. There remains, however, an important group of patients, primarily comprising those with CHD and IPAH, who depend on HLT as their only hope for survival. Presently, these patients are disadvantaged by longer waits and higher waiting list mortality compared to those listed for single organ transplantation despite comparable post-transplant outcomes. We must address the need for equity by acknowledging the conflict between treating 2 or 3 patients with the same organs that with HLT go to 1 patient. These individuals share a right to life, and nuanced prioritization criteria tailored to HLT and informed appropriately by biopsychosocial and economic factors, are now required.

## Financial support

This study is part funded by the National Institute for Health and Care Research (NIHR) Blood and Transplant Research Unit in Organ Donation and Transplantation (NIHR203332), a partnership between NHS Blood and Transplant, the University of Cambridge, and Newcastle University. The views expressed are those of the author(s) and not necessarily those of the NIHR, NHS Blood and Transplant, or the Department of Health and Social Care. Dr Louise Coats is funded by an NIHR Senior Clinical Practitioner Research Award.

## Conflicts of Interest statement

The authors declare the following financial interests/personal relationships, which may be considered as potential competing interests: Louise Coats reports financial support was provided by the National Institute for Health and Care Research. Louise Coats reports a relationship with the National Institute for Health and Care Research that includes: funding grants. If there are other authors, they declare that they have no known competing financial interests or personal relationships that could have appeared to influence the work reported in this paper.
